# Ulcerative Enteritis in the Horse1Read before the Fifteenth Annual Meeting of the Iowa State Veterinary Medical Association, Cedar Rapids, Iowa, January 14 and 15, 1903.

**Published:** 1903-04

**Authors:** G. L. Buffington

**Affiliations:** Baxter, Iowa


### ULCERATIVE ENTERITIS IN THE HORSE.[1]

By G. L. Buffington, D.V.M.,
BAXTER, IOWA.

The essential organs of digestion, the stomach and intestines, being charged with the complex process of converting food into nutriment which may be assimilated, and thus used in building up and maintaining all tissues and organs of the animal body, are, of necessity, very complex in their organization. That they are capable of disintegrating and separating the various foods into material which can be used in building up all parts of the animal, by supplying it with the required nutriment for maintaining heat and energy, is evidence that they have a vast amount of work to perform and that the physiological process is very complex. The anatomical construction also favors the physiological process. The great length of the alimentary canal, the irregular dilations and sacculations and the longitudinal bands with which it is traversed, give to it a very extensive surface to present to the alimentary matter in its onward passage through the canal. It also tends to stay its progress until all the material has been subjected to the digestive fluids, so that nothing can escape which may be utilized for nutrition. The normal peristaltic movements also assist digestion by increasing the secretions and producing a more thorough mixture of the alimentary matters.

Considering, then, that all the functions of the alimentary canal must be performed in a normal condition in order to insure perfect health, and that it is compelled under so many circumstances and

[1] Read before the Fifteenth Annual Meeting of the Iowa State Veterinary Medical Association, Cedar Rapids, Iowa, January 14 and 15, 1903.

environments to carry on this process of digestion and assimilation under abnormal conditions, it is not strange that we find the digestive organs of the horse subject to many diseases. In this view we may regard the disease as a transformation from the physiological to the pathological conditions brought about by some abnormal condition of the food or environment, or by infection.

We wish to consider briefly but one of the many diseases of the digestive organs of the horse. Ulcerative enteritis is so closely related to some other intestinal affections, that it is often difficult to distinguish between them.

Symptoms. The symptoms are often obscure at the beginning, but soon manifest themselves in such a decided manner, that the attendant is convinced he has a very serious case to deal with. The first symptom usually observed is refusal of all food, or if he is at pasture, where this would not so readily be observed, a profuse diarrhœa first attracts the owner's attention. The horse appears dull and languid; a general tucked-up appearance is observed; the buccal mucous membrane is usually red, and in cases of a subacute type the membrane is ulcerated, as is also the tongue; petechiæ cover the nasal mucous membrane and sore spots sometimes appear under the alæ of the nostrils, though this is not a constant symptom. There is usually a foul odor in the mouth; the pulse is accelerated and feeble; the temperature is not usually high, ranging from 102° to 104°, or perhaps as high as 105°. Diarrhœa often sets in early in the disease, and, if the inflammation is not intense enough to destroy life quickly, the evacuations become liquid and dark-colored, and in some cases run from the anus in a stream without any expulsive effort on the part of the horse; borborygmus is present; a craving for salt was observed in one case, though it refused all food. As the disease progresses the symptoms are indicative of the most intense pain. The animal will roll around in every conceivable shape. I have observed them to roll upon the back and remain in that position for some time as though it afforded some relief. The veterinarian called at this time might mistake the symptoms for those of spasmodic colic, but an examination of the pulse, which is now very rapid and feeble, will convince him that he has something much more serious than colic to deal with, as in colic the pulse is normal, or nearly so. The character of the pain is also different. In colic it is subject to remissions, while in this form of enteritis it is constant. Manipulation or pressure over the abdomen will reveal the watery contents of the bowels, and also will often cause the animal to evince pain.

Course of the Disease. The course of the disease may vary from one to three days in acute to as many weeks in subacute cases. A large percentage of cases prove fatal, but when recovery does take place it is very slow.

Treatment. A considerable number of horses suffering from this disease are beyond the reach of medical treatment before the veterinarian is called, and I believe most of them are hopeless from the outset. Where the symptoms are not so aggravated but that there is some hope of recovery, beneficial effects may be expected from the application to the abdomen of blankets wrung out of hot water and these covered with dry ones. Opiates are to be given to relieve the pain and quiet the action of the bowels. If diarrhœa is established, subnitrate of bismuth, prepared chalk, gentian, and ginger, along with opiates. In one subacute case which had refused all food for six days and then began purging, I gave the following prescription: Prepared chalk, gentian powder, subnitrate of bismuth, potassium chlorate, each four drachms; powdered ginger, two drachms.

This was mixed with a quart of milk and three or four eggs, and then four drachms of tincture of chloride of iron added and given three times a day. The patient began to improve in about two days and made a complete, but slow, recovery.

Post-mortem Appearances. The lesions on post-mortem examination are well defined and very severe. No tympanites is present, but the intestinal canal is filled with a dark-colored, watery fluid; in no case have I observed any trace of any solid excrement in any part of the intestinal canal. The wall of the large colon and cæcum is thickened to four or five times its normal thickness, though this thickening is not uniform in all parts of the intestinal walls. Some portions will be necrosed and of a green or black color. The internal surface of the colon and cæcum is dotted with ulcers of various sizes up to about one-half inch in diameter. These ulcers are irregular in outline with ragged margins, and are sometimes raw and sometimes covered with yellowish exudate which easily scrapes off, leaving a raw surface.

I observed one case in which the ulcerations were more uniform in outline and much smaller than those above described. They were small circular ulcers, and the mucous membrane of both colon and cæcum was studded with them. The small intestine shows inflammation of a catarrhal nature, but I have as yet observed no ulcers in that region. The stomach is somewhat reddened, but I have not seen any indication of much inflammation in that organ.

In some cases there is ulceration of the mouth and tongue. The mesenteric lymphatic glands are somewhat softened. The blood is fluid, dark-colored, and very scant.

Etiology. I have very little of value to offer on this very important phase of the subject. The cause is as obscure as the treatment is unsatisfactory. It attacks horses of all ages, under circumstances and environments which we would think ought to be favorable to health.

It was observed in a large three-year-old Clydesdale mare last September, that had run in a good blue-grass pasture all summer with a number of other horses and colts, all of which continued in the best of health. In another case, a twelve-year-old, well-bred, large, driving mare that had been running in pasture all summer suckling a colt, became affected on October 11th, and was dead at noon the next day. She was seen on October 10th and was not noticed to be sick, but was found the next morning scouring profusely and died in about thirty hours; so she could not have been sick more than about forty-eight hours at the most.

Another case, pure-bred weanling Clydesdale colt, had been running in good pasture with other colts in daytime during the fall and early winter. They were put in the stable at night and fed oats. On December 4th this colt was observed to be dull and stupid and not inclined to eat, but did not seem much sick until four days later, when I saw him and found a temperature of $104\frac{4}{5}°$, labored respiration, and quick, feeble pulsation, but not suffering much pain; bowels moving about normal. The following day he began to manifest considerable pain and rapidly grew worse, and on the evening of the next day died. There was no discharge of fæces during the last two days, but on post-mortem examination the characteristic fluid condition of the intestinal contents and absence of any solid material, together with the usual lesions of an inflamed and ulcerated colon and cæcum, determined the seat of the disease.

Another case was a seven-year-old mare that had been kept up on dry feed and worked at light work on the farm. This was but a mild case and recoverd.

The cause is probably to be found in a fungus or mould of some kind which develops on the grass or weeds, though this, so far as I know, has never been demonstrated. The past summer and fall were excessively moist, a condition which would favor the growth of fungus or mould, and it seems to have been more prevalent the past fall than heretofore. At least, it is the first time it has come under my observation.

